# Supplementary material for: Cephalosporin translocation across enterobacterial OmpF and OmpC channels, a filter across the outer membrane
Source: Commun Biol. 2022 Oct 5;5:1059. doi: 10.1038/s42003-022-04035-y (PMC9534850; doi:10.1038/s42003-022-04035-y)
Supplement: Supplementary file 2 — Supplementary Information [file 42003_2022_4035_MOESM2_ESM.pdf]

SUPPLEMENTARY INFORMATION

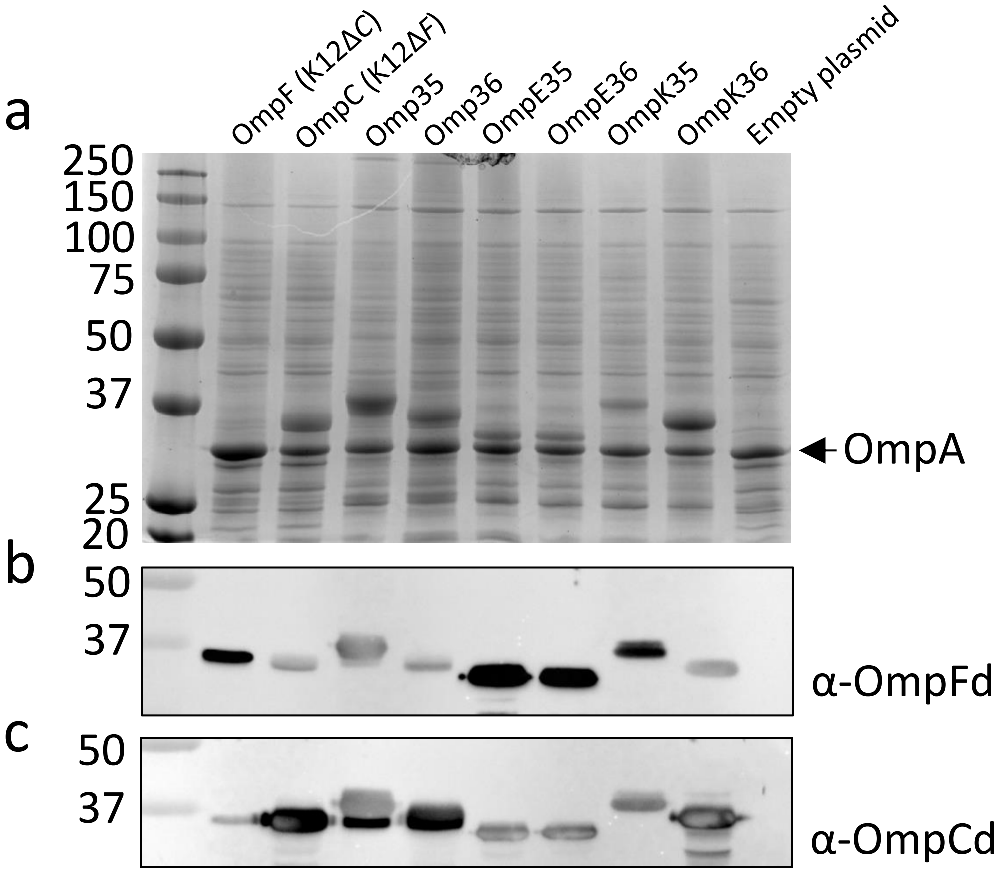

Supplementary Figure 1. SDS-PAGE and immunoblot analysis of the outer membrane proteins prepared from W3110*ΔompC*, W3110*ΔompF* and W3110*ΔFC* expressing individual porin orthologues. Expression of porin orthologues was induced with 0.02 % L-arabinose at 37°C for 2 h. Samples equivalent to 0.2 OD units were applied to 10 % SDS-PAGE after heating at 100°C for 5 min in Laemmli buffer. **a**, Total outer membrane proteins were stained with Coomassie Brilliant Blue R250. Porin bands are found above OmpA (arrowhead). **b** and **c**, Porin orthologues were immunodetected with antibodies directed against denatured OmpF (**b**, OmpFd) or OmpC (**c**, OmpCd). One could note that Omp36 and OmpK36 on one hand, and OmpF, OmpE35, OmpE36 and OmpK35 on the other hand showed strong reactivity with α-OmpCd and α-OmpFd antibodies, respectively. Omp35 showed modest reactivity with both α-OmpFd and α-OmpCd. MW (kDa) are indicated on the left. Western-blot are from uncropped images.

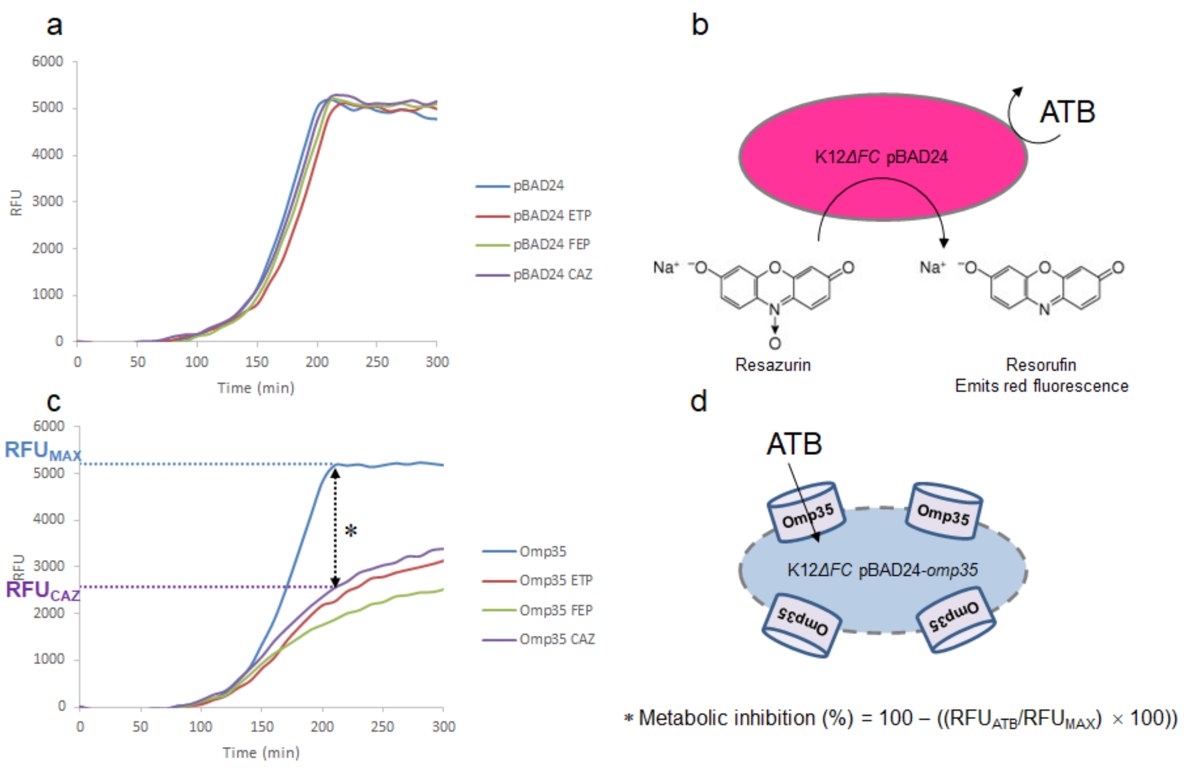

Supplementary Figure 2. Resazurin-reduction based assay to evaluate β-lactam permeability through individual porins. In the absence of antibiotics, actively metabolizing bacterial cells are able to reduce blue resazurin into red resofurin, which emits fluorescence at 590 nm (RFU_{MAX}). Prior to the assay, the final concentration of each antibiotic was determined as to the maximal concentration that yields negligible metabolic inhibition for W3110Δ*FC* transformed with the empty vectors (**a**, **b**). Then, the metabolic inhibition observed for W3110Δ*FC* strains expressing individual porins in the presence of each β- antibiotic was calculated (RFU_{ATB}) (**c**, **d**). a and c show raw data of resofurin fluorescence (RFU) relatively to time (min). b and d are schematic representations of the assay.

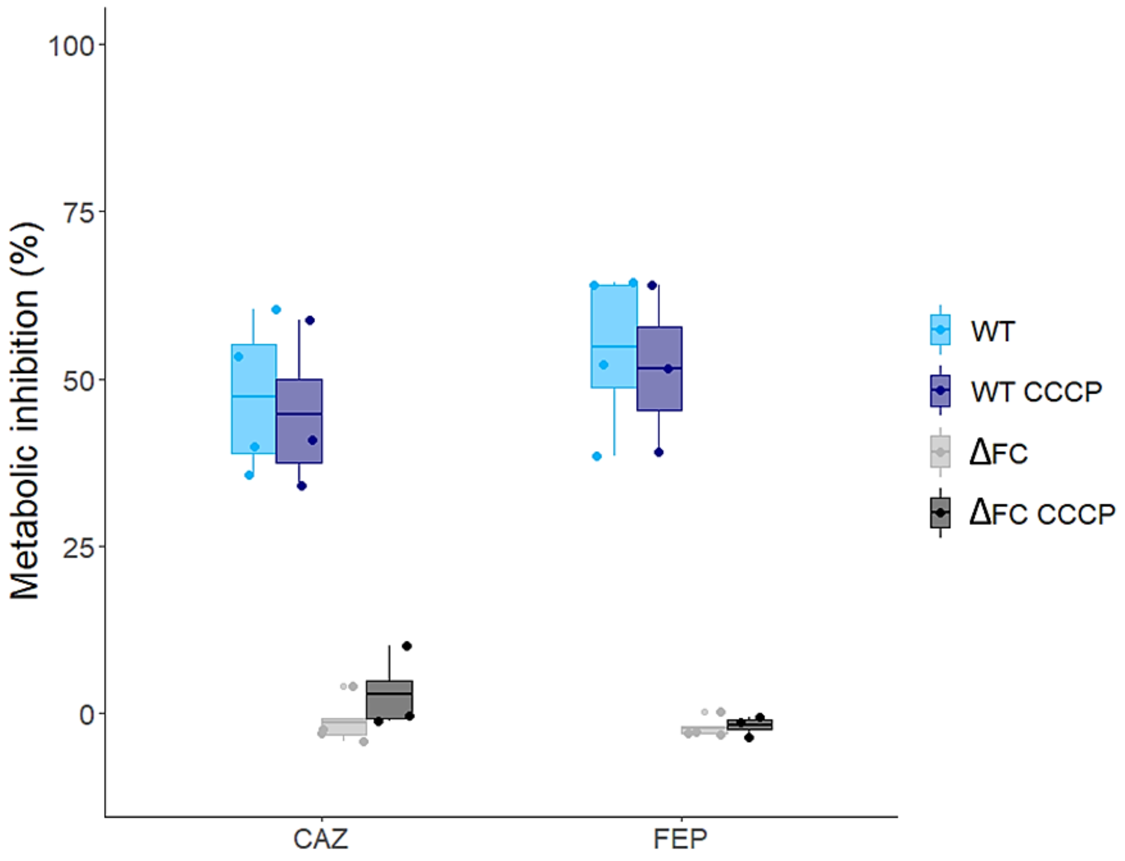

Supplementary Figure 3. Impact of drug efflux on the metabolic inhibition caused by CAZ and FEP. Metabolic inhibition was assayed by using the resazurin-reduction based assay as described in Materials and Methods. WT (blue boxes) and porinless (ΔFC, grey boxes) strains of *E. coli* W3110 were incubated with CAZ or FEP without or with CCCP. Drug efflux was chemically inhibited in the presence of CCCP. The absence of drug efflux does not significantly affect the activity of FEP and CAZ. Results without and with CCCP were obtained respectively from n=4 and n=3 biologically independent experiments. Results are shown with boxplots where the boxes range from the first quartile to the third quartile of the distribution, the median is indicated by a line across the box and the whiskers extend to the most extreme data points.

ceftazidime

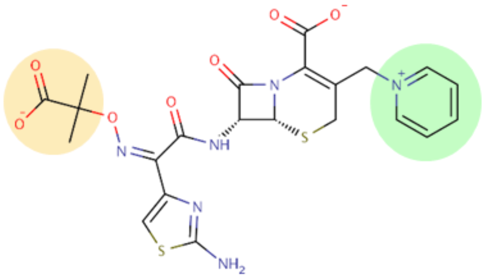

MW:	548 Da
Charges:	1+; 2 -
LogP:	- 4.55
Dipole moment:	25.5 $D_x y$
Rotable bounds:	8
Accumulation:	5 min: 8.9 / 3.0
$OmpF^+/OmpC^+$	15 min: 13.1 / 6.0
$x10^4$ molecules/cellule	30 min: 13.1 / 10.4

cefepime

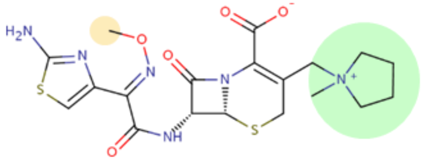

MW:	480 Da
Charges:	1+; 1 -
$ClogD_{7.4}$:	- 3.68
Dipole moment:	17.21 $D_x y$
Rotable bounds:	7
Accumulation:	5 min: 6.3 / 6.9
$OmpF^+/OmpC^+$	15 min: 10.4 / 11.1
$x10^4$ molecules/cellule	30 min: 13.4 / 13.4

ticarcillin

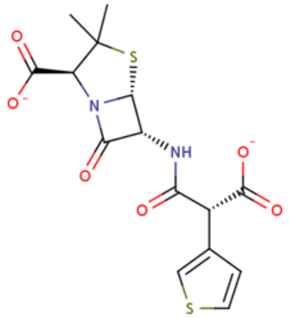

MW:	384 Da
Charges:	2 -
$ClogD_{7.4}$:	- 5.97
Dipole moment:	9.05 $D_x y$
Rotable bounds:	5
Accumulation:	ND

ertapenem

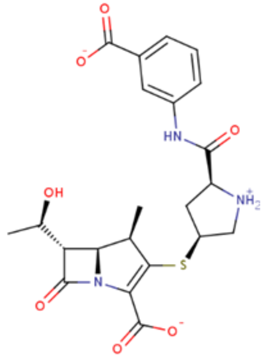

MW:	476 Da
Charges:	1+; 2 -
$ClogD_{7.4}$:	- 6.9
Dipole moment:	26.92 $D_x y$
Rotable bounds:	7
Accumulation:	ND

cefotaxime

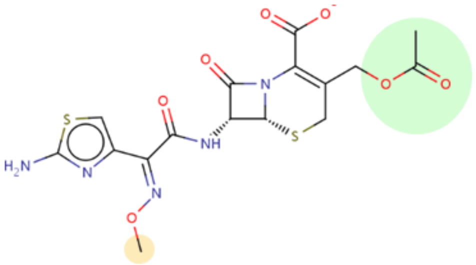

MW:	455 Da
Charges:	1+; 2 -
$ClogD_{7.4}$:	- 4.2
Dipole moment:	19.81 $D_x y$
Rotable bounds:	8
Accumulation:	5 min: 5.3 / 4.8
$OmpF^+/OmpC^+$	15 min: 8.0 / 8.8
$x10^4$ molecules/cellule	30 min: 7.9 / 8.6

piperacillin

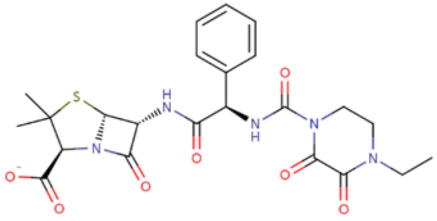

MW:	518 Da
Charges:	1 -
$ClogD_{7.4}$:	- 3.64
Dipole moment:	31.19 $D_x y$
Rotable bounds:	6
Accumulation:	ND

meropenem

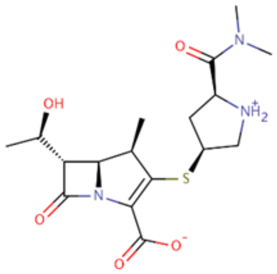

MW:	383 Da
Charges:	1+; 1 -
$ClogD_{7.4}$:	- 4.5
Dipole moment:	30.95 $D_x y$
Rotable bounds:	5
Accumulation:	ND

Supplementary Figure 4. Chemical structures and molecular properties of the tested compounds.

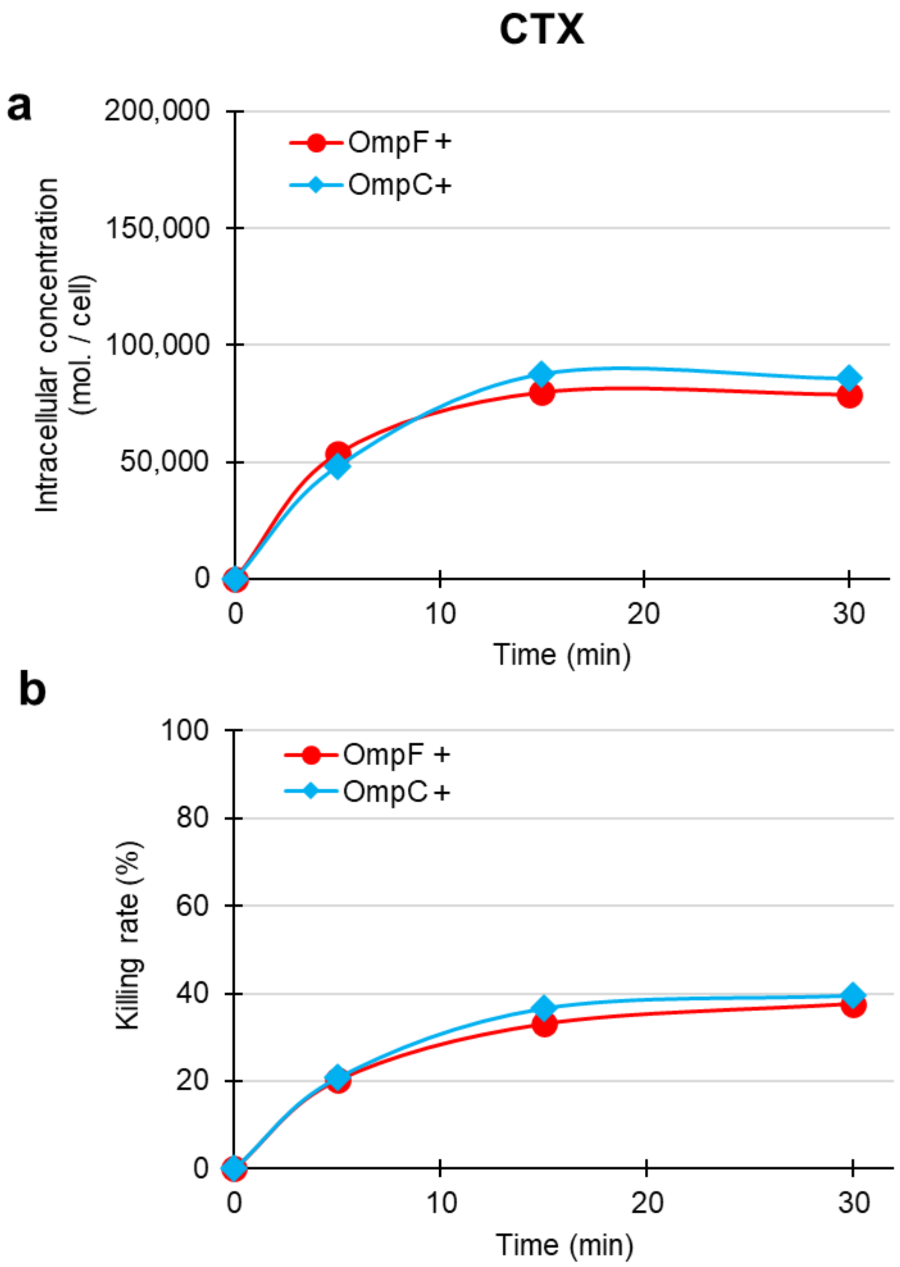

Supplementary Figure 5. Intracellular accumulation and killing rates of CTX in *E. coli* W3110*ΔompC* and W3110*ΔompF*. Intracellular concentrations were obtained from incubation of bacteria with CTX at 16 µg/ml. Bacterial suspensions were sampled at 5, 15 and 30 minutes for LC-MS/MS analysis of the intracellular concentrations (**a**) and CFU/killing rates determination (**b**). The data plotted are means obtained from two independent assays performed in triplicate.

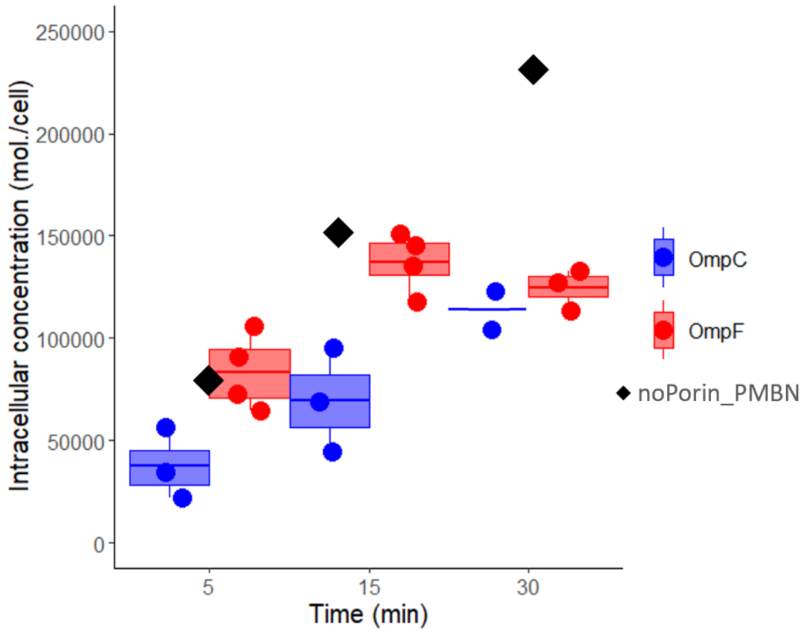

Supplementary Figure 6. Intracellular accumulation of CAZ in *E. coli* W3110*ΔompC* (red boxplot), W3110*ΔompF* (blue boxplot) and W3110*ΔCΔF* incubated with the permeabilizer Polymyxin B nonapeptide (PMBN, black diamonds). Intracellular concentrations were obtained from incubation of bacteria with CAZ at 16 µg/ml without and with PMBN at 102.4 µg/ml. Bacterial suspensions were sampled at 5, 15 and 30 minutes for CFU determination and LC-MS/MS analysis of the intracellular concentrations. Accumulation is reported in a number of antibiotic molecules per CFU. The experiment with W3110*ΔCΔF* + PMBN was performed in triplicate and results with W3110*ΔompC* and W3110*ΔompF* were obtained from three independent experiments carried out in triplicate. Results are shown with boxplots where the boxes range from the first quartile to the third quartile of the distribution, the median is indicated by a line across the box and the whiskers extend to the most extreme data points.

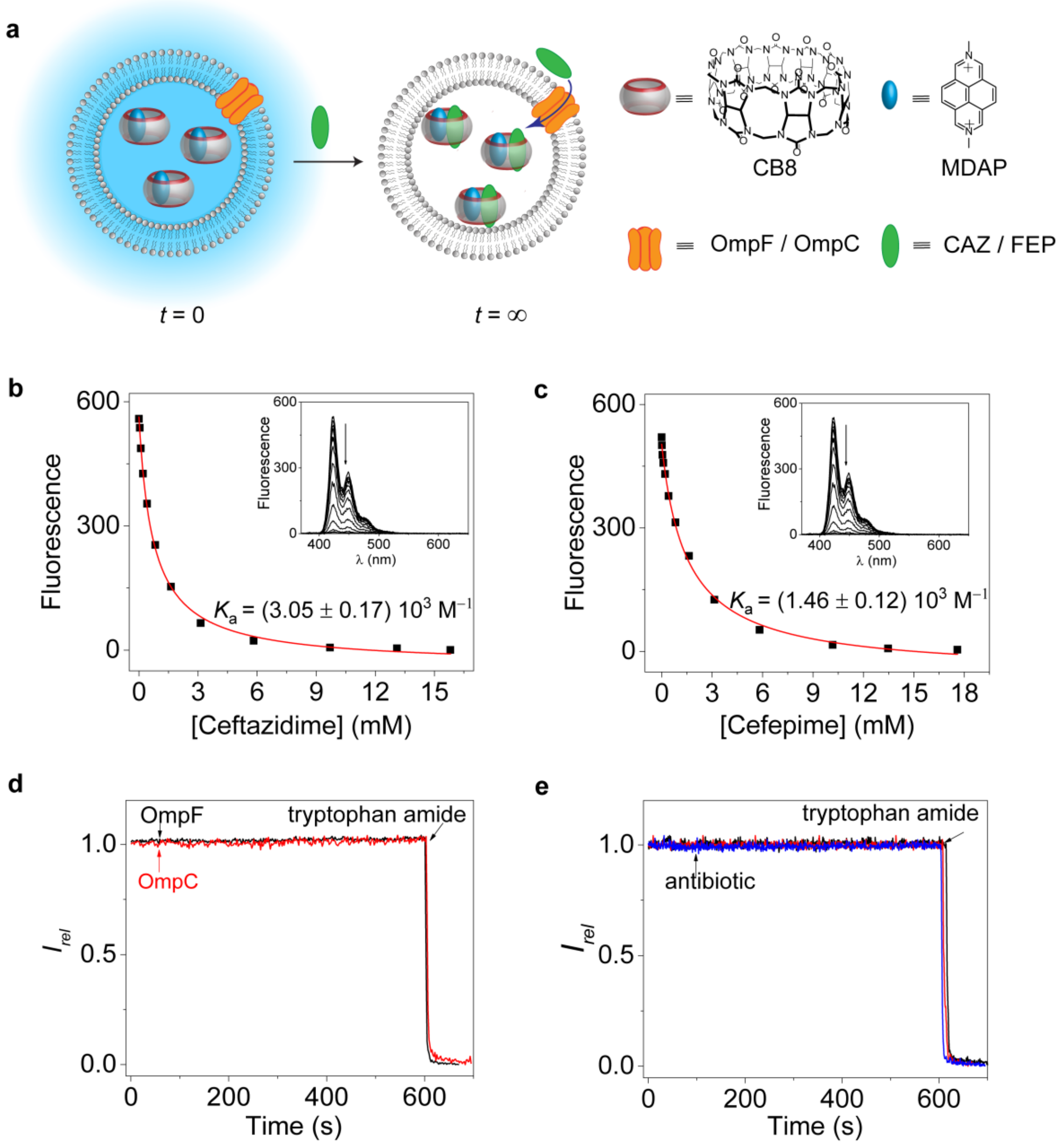

Supplementary Figure 7. a, Working principle of the fluorescent artificial receptor membrane assay, FARMA. The membrane-impermeable fluorescent receptor CB8/MDAP is encapsulated into proteoliposomes containing the porin (left). After analyte addition, antibiotic translocation through the porin (OmpF or OmpC) into the liposome takes place, a ternary CB8/MDAP/antibiotic complex is formed, and a fluorescence quenching results (middle). Chemical structures of the macrocyclic host and the fluorescent dye are shown (right). **b**, **c** and **d**, Fluorescence titrations (λ_{ex}= 339 nm, λ_{em}= 422 nm) with CB8/MDAP (1/1 µM) to determine the binding constants (K_a) with CAZ (b), FEP (c) and CTX (d) in 10 mM Hepes, pH 7.0. The inserts show the respective fluorescence spectra. e, Changes in the fractional MDAP emission (λ_{ex}= 339 nm, λ_{em}= 422 nm) of CB8/MDAP-loaded LUVs (15 µM phospholipids in 10 mM Hepes, pH 7.0) upon addition of first OmpF (45 nM, black trace) or OmpC (45 nM, red trace)

and subsequently tryptophan amine (25 µM). for calibration. The absence of a change in fluorescence emission intensity after porin addition demonstrates that the reporter pair does not escape through the channel. f, Changes in the fractional MDAP emission (λ_{ex} = 339 nm, λ_{em} = 422 nm) of CB8/MDAP-loaded LUVs upon the addition of first CAZ (5 mM, black trace), FEP (5 mM, red trace), or CTX (5 mM, blue trace), and, subsequently, tryptophan amine (25 µM). The absence of changes in the fluorescence emission intensity confirm that the antibiotics do not permeate across the vesicular membrane.

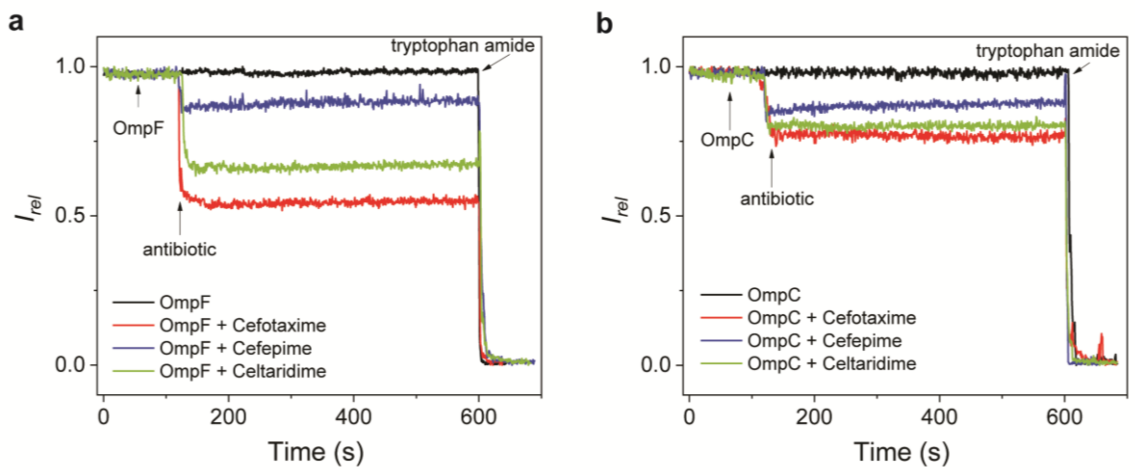

Supplementary Figure 8. Changes in the fractional MDAP emission (λ_{ex}= 339 nm, λ_{em}= 422 nm) of CB8/MDAP-loaded liposomes (15 µM phospholipid in 10 mM Hepes, pH 7.0) upon addition of **a**, OmpF or **b**, OmpC (45 nM, at 60 s), antibiotic (26 µM, at 120 s) and tryptophan amine (25 µM, at 600s) for calibration.

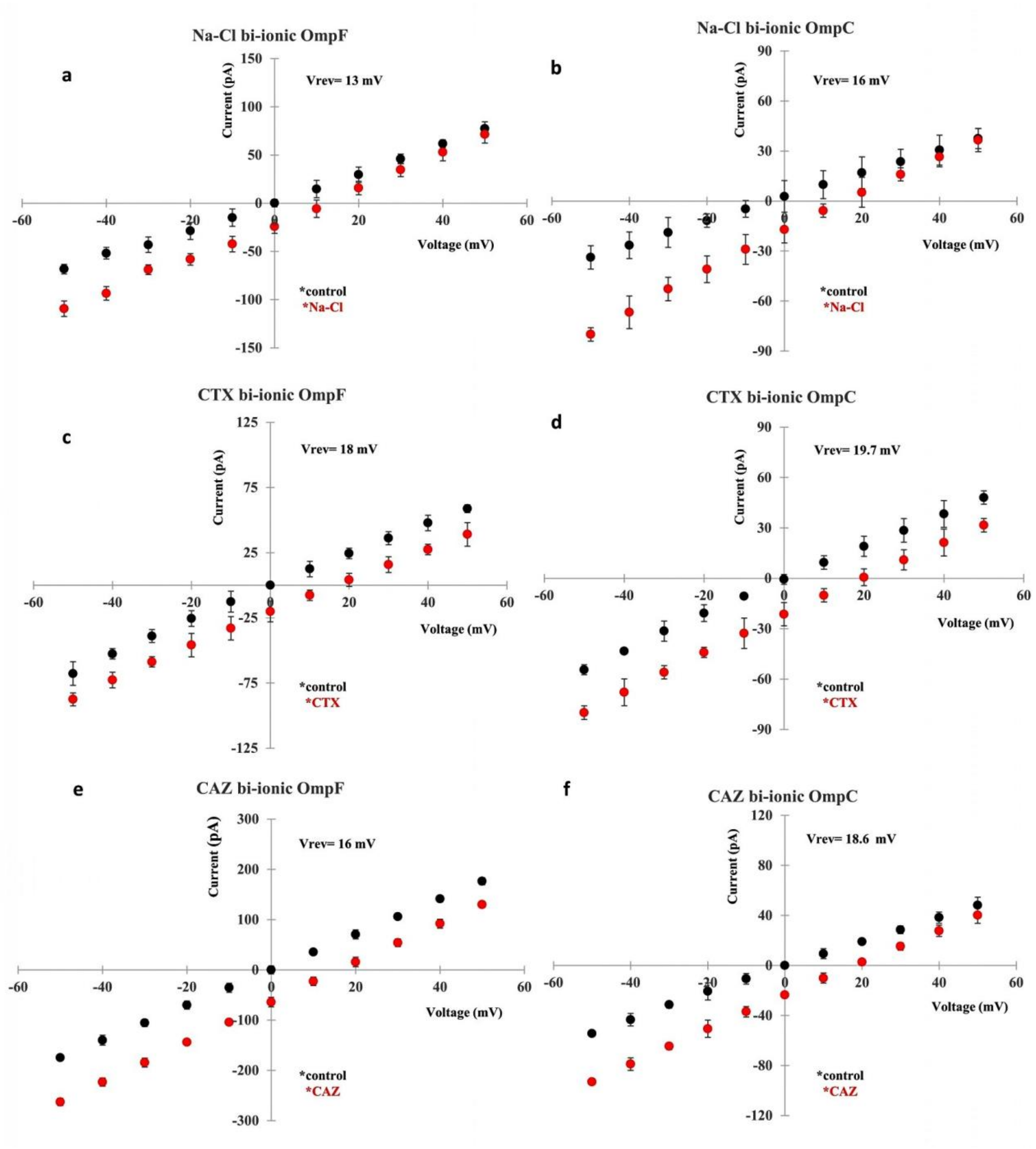

Supplementary Figure 9. Selected ion current vs. applied voltage (I/V) curves from bilayers containing reconstituted OmpF/OmpC channel with NaCl, CAZ or CTX respectively under bi-ionic conditions. Both antibiotics are negatively charged at pH 7. OmpF Bi-ionic current voltage recording with symmetrical 30 mM NaCl (a), CTX (c), CAZ (e) *cis*/*trans* (control (●)) and asymmetrical (80/30 mM NaCl/CTX/CAZ *cis*/*trans* (●). OmpC Bi-ionic current voltage recording with symmetrical 30 mM NaCl (b), CTX (d), CAZ (f) cis/trans (control (●)) and asymmetrical (80/30 mM NaCl/CTX/CAZ *cis*/*trans* (●). The zero-current membrane potentials V_{rev} in the presence of different concentrations of the electrolyte on both sides of the planer lipid bilayer with OmpF and OmpC inserted were determined and reported in Table 1. The pH range throughout the study was maintained at pH= 7±0.5 for all substrates.

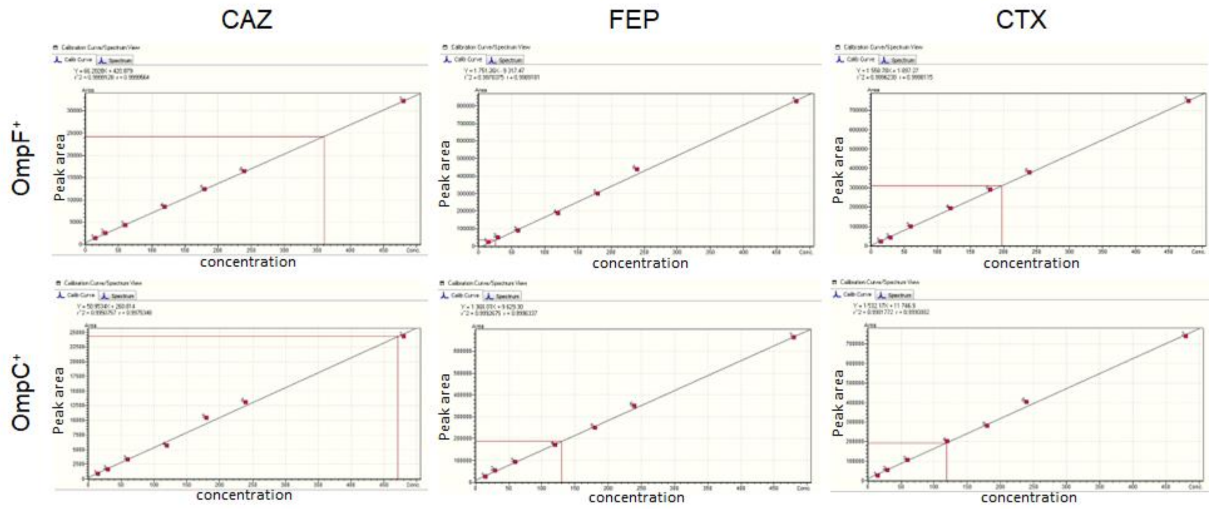

Supplementary Figure 10. Antibiotic calibrations for LC MS/MS analysis.

Supplementary Table 1. MICs (µg/ml) were determined in Mueller Hinton 2 broth by the microdilution method.

Strain	W3110	W3110 Δ*FC*		
Plasmid	pBAD24	pBAD24	pBAD24-Omp F orthologues	pBAD-OmpC orthologues
Ertapenem	0.004	0.125	0.125	0.125
Meropenem	0.015	0.125	0.125	0.125
Imipenem	0.125	0.125	0.125	0.125
Cefepime	0.031	0.25	0.25	0.25
Ceftazidime	0.125	0.25	0.25	0.5
Aztreonam	0.031	0.25	0.25	0.25
Cefoxitine	2	16	16	16
Ceftriaxone	0.008	0.063	0.063	0.063
Piperacillin	1	1	1	1
Ticarcillin	2	2	2	64
Ceftaroline	0.031	0.063	0.063	0.125
Chloramphénicol	4	8	8	8

Supplementary Table 2. Transport properties of OmpF and OmpC channels revealed by electrophysiology.

Pore	Substrate 30mM cis/trans	Conductance pS	Bulk Conductance mS/cm
OmpF	Sodium Chloride	272 ± 35 (n=6)	3.3 ± 0.3
	Ceftazidime -Sodium	182 ± 44 (n=3)	1.9 ± 0.2
	Cefotaxime -Sodium-	160 ± 70 (n=3)	1.8 ± 0.2
OmpC	Sodium Chloride	140 ± 30 (n=3)	3.3 ± 0.3
	Ceftazidime -Sodium	110 ± 30 (n=3)	1.9 ± 0.2
	Cefotaxime -Sodium	108 ± 40 (n=6)	1.8 ± 0.2

Experimental and calculated conductance (±SD) of an OmpF and OmpC trimer at low ionic strength under bi-ionic conditions.
