# Supplementary material for: Cephalosporin translocation across enterobacterial OmpF and OmpC channels, a filter across the outer membrane
Source: Commun Biol. 2022 Oct 5;5:1059. doi: 10.1038/s42003-022-04035-y (PMC9534850; doi:10.1038/s42003-022-04035-y)
Supplement: Supplementary file 5 — Reporting Summary [file 42003_2022_4035_MOESM5_ESM.pdf]

nature research

Corresponding author(s): Jean-Marie PAGES

Last updated by author(s): Sep 14, 2022

Reporting Summary

Nature Research wishes to improve the reproducibility of the work that we publish. This form provides structure for consistency and transparency in reporting. For further information on Nature Research policies, see our Editorial Policies and the Editorial Policy Checklist.

Statistics

For all statistical analyses, confirm that the following items are present in the figure legend, table legend, main text, or Methods section.

n/a	Confirmed	
☐	☒	The exact sample size (*n*) for each experimental group/condition, given as a discrete number and unit of measurement
☐	☒	A statement on whether measurements were taken from distinct samples or whether the same sample was measured repeatedly
☐	☒	The statistical test(s) used AND whether they are one- or two-sided<br>*Only common tests should be described solely by name; describe more complex techniques in the Methods section.*
☒	☐	A description of all covariates tested
☐	☒	A description of any assumptions or corrections, such as tests of normality and adjustment for multiple comparisons
☐	☒	A full description of the statistical parameters including central tendency (e.g. means) or other basic estimates (e.g. regression coefficient) AND variation (e.g. standard deviation) or associated estimates of uncertainty (e.g. confidence intervals)
☐	☒	For null hypothesis testing, the test statistic (e.g. *F*, *t*, *r*) with confidence intervals, effect sizes, degrees of freedom and *P* value noted<br>*Give P values as exact values whenever suitable.*
☒	☐	For Bayesian analysis, information on the choice of priors and Markov chain Monte Carlo settings
☒	☐	For hierarchical and complex designs, identification of the appropriate level for tests and full reporting of outcomes
☐	☒	Estimates of effect sizes (e.g. Cohen's *d*, Pearson's *r*), indicating how they were calculated

Our web collection on statistics for biologists contains articles on many of the points above.

Software and code

Policy information about availability of computer code

Data collection	n/a
Data analysis	n/a

For manuscripts utilizing custom algorithms or software that are central to the research but not yet described in published literature, software must be made available to editors and reviewers. We strongly encourage code deposition in a community repository (e.g. GitHub). See the Nature Research guidelines for submitting code & software for further information.

Data

Policy information about availability of data

All manuscripts must include a data availability statement. This statement should provide the following information, where applicable:

- Accession codes, unique identifiers, or web links for publicly available datasets
- A list of figures that have associated raw data
- A description of any restrictions on data availability

All data supporting this study are available within the article and its Supplementary Information. Source data are available within the Supplementary Data 1 file. All other data are available from the corresponding author on reasonable request.

Field-specific reporting

Please select the one below that is the best fit for your research. If you are not sure, read the appropriate sections before making your selection.

☒ Life sciences ☐ Behavioural & social sciences ☐ Ecological, evolutionary & environmental sciences

For a reference copy of the document with all sections, see nature.com/documents/nr-reporting-summary-flat.pdf

Life sciences study design

All studies must disclose on these points even when the disclosure is negative.

Sample size	Metabolic inhibition percents shown in Figures 1a and 1b were obtained from n = 6, 10, 7, 6, 5, 4 (CAZ) ; 6, 11, 7, 4, 11, 8 (FEP) ; 6, 11, 8, 6, 11, 9 (CTX) ; 3, 3, 5, 3, 7, 6 (PIP) ; 3, 3, 3, 3, 3, 3 (TIC) ; 6, 10, 5, 6, 10, 8 (ETP) ; 6, 11, 6, 6, 11, 9 (MEM) independent experiments with strains expressing Omp35, OmpE35, OmpK35, Omp36, OmpE36, OmpK36, respectively.<br>Metabolic inhibition results in Figures 1a and Supplementary Figure 3 were obtained from n = 2 (Figure 1c), 3 (Supplementary Figure 3, with CCCP) and 4 (Supplementary Figure 3, without CCCP) independent experiments.<br>Boxplot of accumulated concentrations shown in Figures 2 and 3 were obtained during three biologically independent assays (n=3).<br>Pearson's Coefficients of determination (Figure 4) were calculated with n=38 (CAZ) and n=17 (FEP) independent samples.<br>Accumulation and killing results of Supplementary Figure 5 were from 2 biologically independent experiments (n=2).<br>Accumulation results shown in Supplementary Figure 6 were obtained from three biologically independent experiments (OmpF+ and OmpC+) or from one experiment (No porin strain with PMBN).
Data exclusions	Technical replicates from accumulation assays were excluded when their LC-MS/MS peaks were outside the linear part of the calibration curves.
Replication	Accumulation and killing assays shown in Figures 2 and 3 were carried out in triplicate and duplicate (killing) during three independent assays.<br>Accumulation and killing assays shown in Supplementary Figure 5 were carried out in duplicate during two independent assays.<br>Accumulation results shown in Supplementary Figure 6 were obtained from triplicate during three independent assays (OmpF+ and OmpC+) or from triplicate of one assay (porinless+PMBN).<br>Two (Figure 1c) or three to eleven independent assays of metabolic inhibition were performed to obtain the results shown in Figure 1 / Supplementary Figure 3.<br>Minimum Inhibitory concentrations shown in Supplementary Table 1 were obtained three times during three biologically independent assays.<br>Three Multi-channel reversal potential experiments were performed to obtain the mean (± SD) shown in Table 1.<br>Conductance measurements shown in Supplementary Table 2 were repeated three or six times.
Randomization	Not relevant in this study
Blinding	Not relevant in this study

Reporting for specific materials, systems and methods

We require information from authors about some types of materials, experimental systems and methods used in many studies. Here, indicate whether each material, system or method listed is relevant to your study. If you are not sure if a list item applies to your research, read the appropriate section before selecting a response.

Materials & experimental systems

n/a	Involved in the study	
☐	☒	Antibodies
☒	☐	Eukaryotic cell lines
☒	☐	Palaeontology and archaeology
☒	☐	Animals and other organisms
☒	☐	Human research participants
☒	☐	Clinical data
☒	☐	Dual use research of concern

Methods

n/a	Involved in the study	
☒	☐	ChIP-seq
☒	☐	Flow cytometry
☒	☐	MRI-based neuroimaging

Antibodies

Antibodies used	Polyclonal rabbit antibodies directed against the denatured monomers of E. coli OmpF or OmpC.<br>Goat anti-rabbit HRP-conjugated secondary antibodies (Bio-Rad).
Validation	Primary antibodies were validated and used in numerous publications.
